# Clinical Implications of Cancer Genomics: A Call for Papers

**DOI:** 10.1371/journal.pmed.1002016

**Published:** 2016-04-26

**Authors:** The PLOS Medicine Editors

**Affiliations:** The *PLOS Medicine* Editors are Clare Garvey, Thomas McBride, Linda Nevin, Larry Peiperl, Amy Ross, Paul Simpson, and Richard Turner

## Abstract

In this month’s editorial, the *PLOS Medicine* Editors announce an upcoming Special Issue, with Guest Editors Elaine Mardis and Marc Ladanyi, on actionable advances in research on the cancer genome.

PLOS Medicine, the leading open access medical journal, is regularly publishing special issues in order to highlight and encourage progress in especially active and important areas of clinical research [1]. As part of this program, the journal is now inviting submissions of research papers to be considered for publication in a special issue devoted to the clinical implications of cancer genomics, which is planned for publication late in 2016.

We are delighted that two leaders in the field, Prof. Elaine Mardis (McDonnell Genome Institute at Washington University, St. Louis, MO, United States) and Dr. Marc Ladanyi (Memorial Sloan Kettering Cancer Center, New York, NY, US), have agreed to join us as guest editors for this issue, which in addition to original research articles will include commissioned Editorials and Perspectives on key topics in the area.

According to the Global Burden of Disease Cancer Collaboration, an estimated 14.9 million new cases of cancer were diagnosed across 188 countries in 2013, and there were 8.2 million deaths [2]. Given this enormous number of people affected by different types of malignant disease, cancer prevention is a high priority—seeking to reduce the burden of cancers caused by environmental and behavioral factors such as tobacco smoke. Yet the aging of populations across high-income, middle-income, and low-income countries alike is expected to lead to ongoing increases in cancer incidence, substantially expanding the need for and costs of treatment and supportive care in all countries.

Intensive research has led to a radical transformation of cancer treatment in recent decades, with agents targeted to the molecular features of patients’ tumors becoming available for various types of disease and adding to the therapeutic benefits of cytotoxic drugs. However, outcomes vary greatly according to cancer type, disease stage, and other factors. Although very good outcomes of treatment are possible in some diseases, such as early-stage Hodgkin’s lymphoma or testicular cancer, progress has been much slower in improvement of treatment for other types of malignancy, and development of resistance to therapies is a major obstacle. Basic, translational, and clinical research aimed at the creation of new and improved treatments in oncology is therefore an important need.

In the past few years, dramatic reduction in the cost of DNA sequencing and advances in sequencing technologies have enabled key insights into the genomic alterations and somatic mutations that cause some types of cancer and accompany disease progression. The drivers and molecular evolutionary history of different types of cancer are now beginning to be understood, and this molecular information is being used to develop novel, and in many cases tailored, therapies. Open access publication is especially well suited to enabling broad access to findings of such data-rich studies, which is needed to catalyze progress in fast-moving research areas, including oncology [3].

The field of cancer genomics is at an exciting juncture: especially promising results are emerging from immunotherapy and vaccine development programs. The forthcoming special issue of *PLOS Medicine* will focus on this area, and we invite submissions of research papers documenting the acquisition and use of genomic information to improve diagnosis, prognosis, or treatment of cancer. We will consider high-quality translational and clinical studies, and priority will be given to research articles describing novel methodologies or treatments that promise to lead to clinical benefit in common cancers or types of malignant disease in which there is a particular need for new treatment strategies.

We especially welcome submissions in the following areas:

- Characterization of genetic alterations leading to carcinogenesis or disease progression in patients, with clear demonstrated relevance for diagnosis or treatment.
- Cancer immunotherapy—novel approaches to harnessing the immune system to combat common cancers.
- Translational or clinical studies aimed at development of cancer vaccines, especially those informed by tumor genome alterations.
- New approaches based on liquid biopsy for disease detection and monitoring of treatment responses.
- Large-scale data analyses and decision-support tools that promise to be of particular importance in aiding cancer diagnosis, provision of prognostic information, and therapies.

Researchers who would like their work to be considered for this special issue should submit by Friday, 8 July 2016. Authors of submissions not selected for the issue may be offered transfer of their paper to *PLOS ONE*, before or after peer review, with the prospect of inclusion in a Collection on the Clinical Implications of Cancer Genomics, which will include papers published in both journals. Please submit your manuscript at this site: http://journals.plos.org/plosmedicine/s/submit-now.

Authors are not required to send a pre-submission inquiry when submitting a manuscript for this special issue. Please indicate in your cover letter that you would like the full manuscript to be considered for the special issue and, if you would like to inquire about the suitability of a manuscript for consideration, please email the editors via plosmedicine@plos.org.
